# Supplementary material for: Allopurinol attenuates development of Porphyromonas gingivalis LPS-induced cardiomyopathy in mice
Source: PLoS One. 2025 Apr 3;20(4):e0318008. doi: 10.1371/journal.pone.0318008 (PMC11967946; doi:10.1371/journal.pone.0318008)
Supplement: S1 Data — (PDF) [file pone.0318008.s001.pdf]

S1 Data

Allopurinol attenuates development of *Porphyromonas gingivalis* LPS-induced cardiomyopathy in mice

Running title: Oxidative heart stress in periodontitis

Akinaka Morii [1, 2 ¶], Ichiro Matsuo [2, 3¶], Kenji Suita [1], Yoshiki Ohnuki [1], Misao Ishikawa [4], Aiko Ito [5], Go Miyamoto [1, 5], Mariko Abe [1, 5], Takao Mitsubayashi [1, 5], Yasumasa Mototani [1], Megumi Nariyama [6], Ren Matsubara [1, 6], Yoshio Hayakawa [7], Yasuharu Amitani [8], Kazuhiro Gomi [2], Takatoshi Nagano [2], Satoshi Okumura [1*]

[1] Department of Physiology, Tsurumi University School of Dental Medicine, Yokohama 230-8501, Japan
[2] Department of Periodontology, Tsurumi University School of Dental Medicine, Yokohama 230-8501, Japan
[3] Department of Oral and Maxillofacial Surgery, Ibaraki Medical Center Tokyo Medical University, Ibaraki 300-0395, Japan
[4] Department of Oral Anatomy, Tsurumi University School of Dental Medicine, Yokohama 230-8501, Japan
[5] Department of Orthodontology, Tsurumi University School of Dental Medicine, Yokohama 230-8501, Japan
[6] Department of Pediatric Dentistry, Tsurumi University School of Dental Medicine, Yokohama 236-8501, Japan
[7] Department of Dental Anesthesiology, Tsurumi University School of Dental Medicine, Yokohama 230-8501, Japan
[8] Department of Mathematics, Tsurumi University School of Dental Medicine, Yokohama, Japan

Supplemental Figure 1

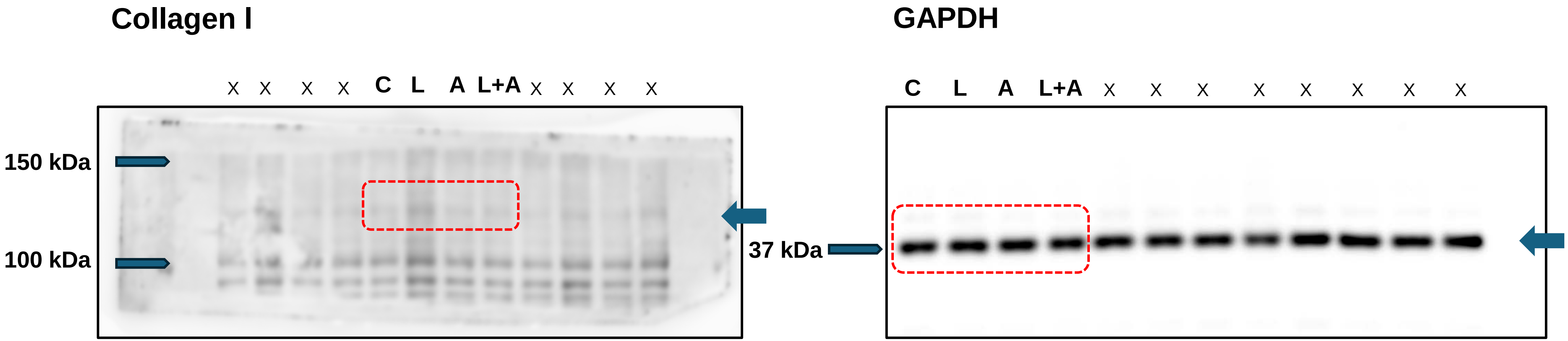

Supplemental Figure 1
Representative full-length immunoblots of **Fig 2C**. The amounts of collagen 1 (left panel) and GAPDH (right panel) are shown. The box outlined in red in each panel, indicated by the arrow, corresponds to the cropped part of the blot. C: control, L: PG-LPS, A: allopurinol, L+A: PG-LPS + allopurinol

Supplemental Figure 2

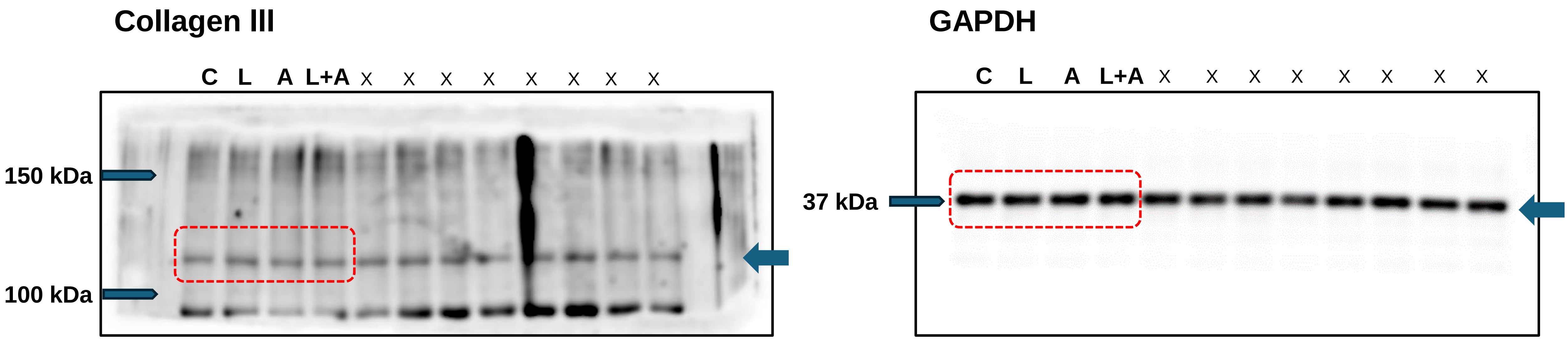

Supplemental Figure 2
Representative full-length immunoblots of **Fig 2D**. The amounts of collagen 3 (left panel) and GAPDH (right panel) are shown. The box outlined in red in each panel, indicated by the arrow, corresponds to the cropped part of the blot. C: control, L: PG-LPS, A: allopurinol, L+A: PG-LPS + allopurinol.

Supplemental Figure 3

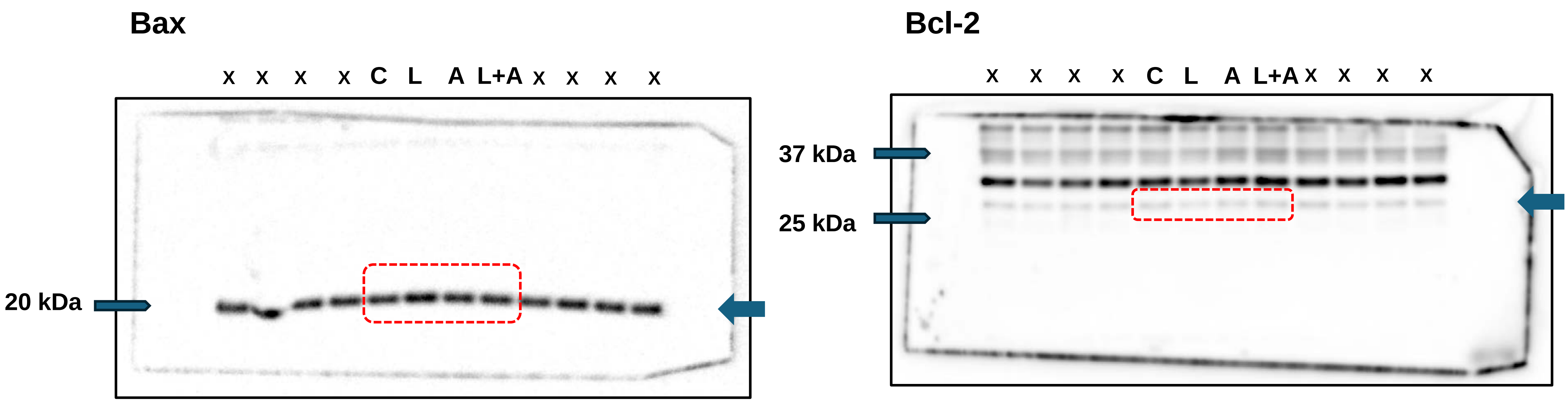

Supplemental Figure 3
Representative full-length immunoblots of **Fig 3C**. The amounts of Bax (left panel) and Bcl-2 (right panel) are shown. The box outlined in red in each panel, indicated by the arrow, corresponds to the cropped part of the blot. C: control, L: PG-LPS, A: allopurinol, L+A: PG-LPS + allopurinol

Supplemental Figure 4

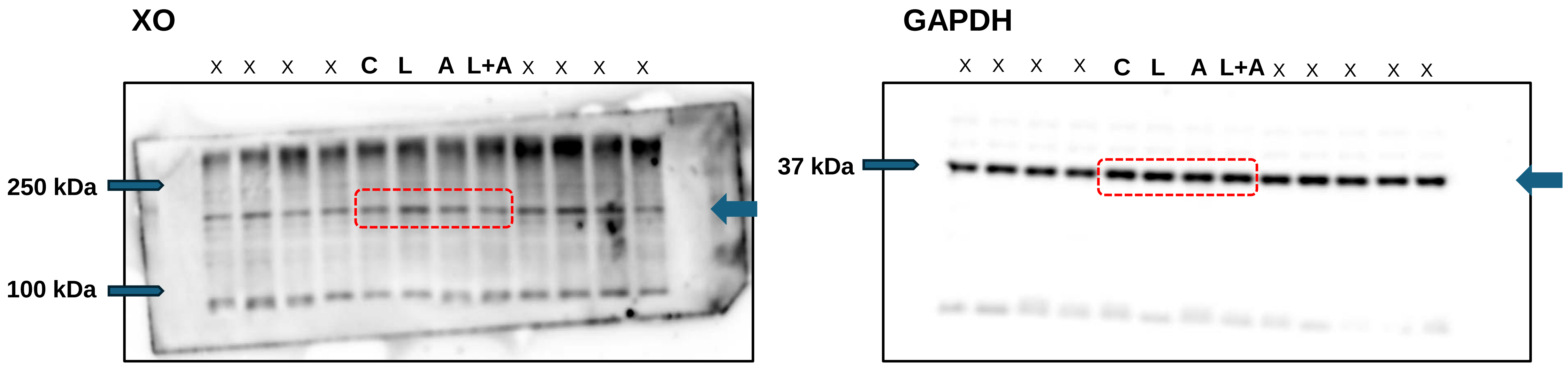

Supplemental Figure 4
Representative full-length immunoblots of **Fig 4C**. The amounts of XO (left panel) and GAPDH (right panel) are shown. The box outlined in red in each panel, indicated by the arrow, corresponds to the cropped part of the blot. C: control, L: PG-LPS, A: allopurinol, L+A: PG-LPS + allopurinol

Supplemental Figure 5

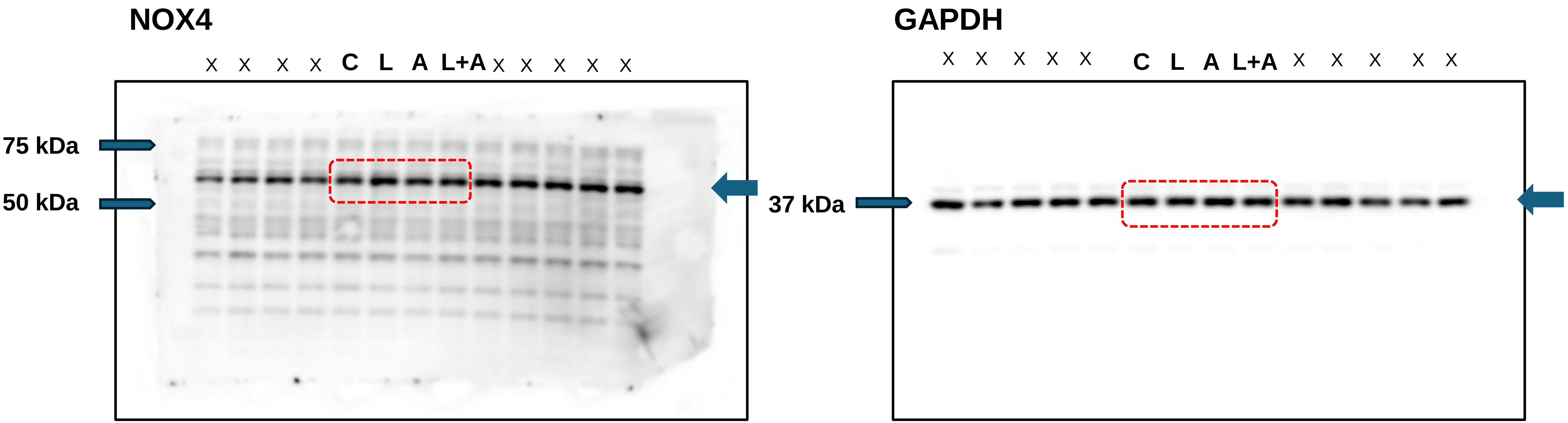

Supplemental Figure 5
Representative full-length immunoblots of **Fig 5A.** The amounts of NOX4 (left panel) and GAPDH (right panel) are shown. The box outlined in red in each panel, indicated by the arrow, corresponds to the cropped part of the blot. C: control, L: PG-LPS, A: allopurinol, L+A: PG-LPS + allopurinol

Supplemental Figure 6

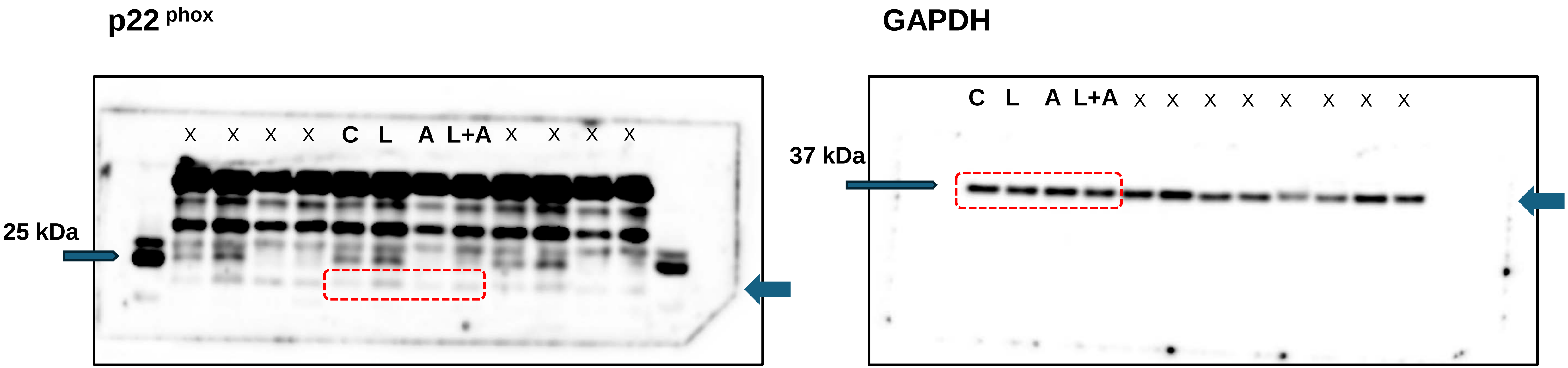

Supplemental Figure 6
Representative full-length immunoblots of **Fig 5B**. The amounts of p22^{phox} (left panel) and GAPDH (right panel) are shown. The box outlined in red in each panel, indicated by the arrow, corresponds to the cropped part of the blot. C: control, L: PG-LPS, A: allopurinol, L+A: PG-LPS + allopurinol

Supplemental Figure 7

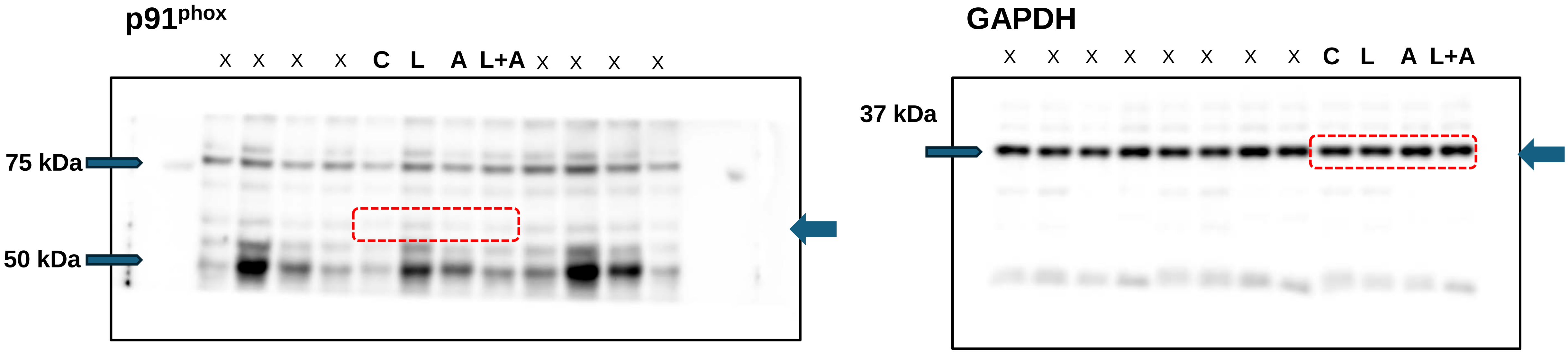

Supplemental Figure 7

Representative full-length immunoblots of **Fig 5C**. The amounts of p91[phox] (left panel) and GAPDH (right panel) are shown. The box outlined in red in each panel, indicated by the arrow, corresponds to the cropped part of the blot. C: control, L: PG-LPS, A: allopurinol, L+A: PG-LPS + allopurinol

Supplemental Figure 8

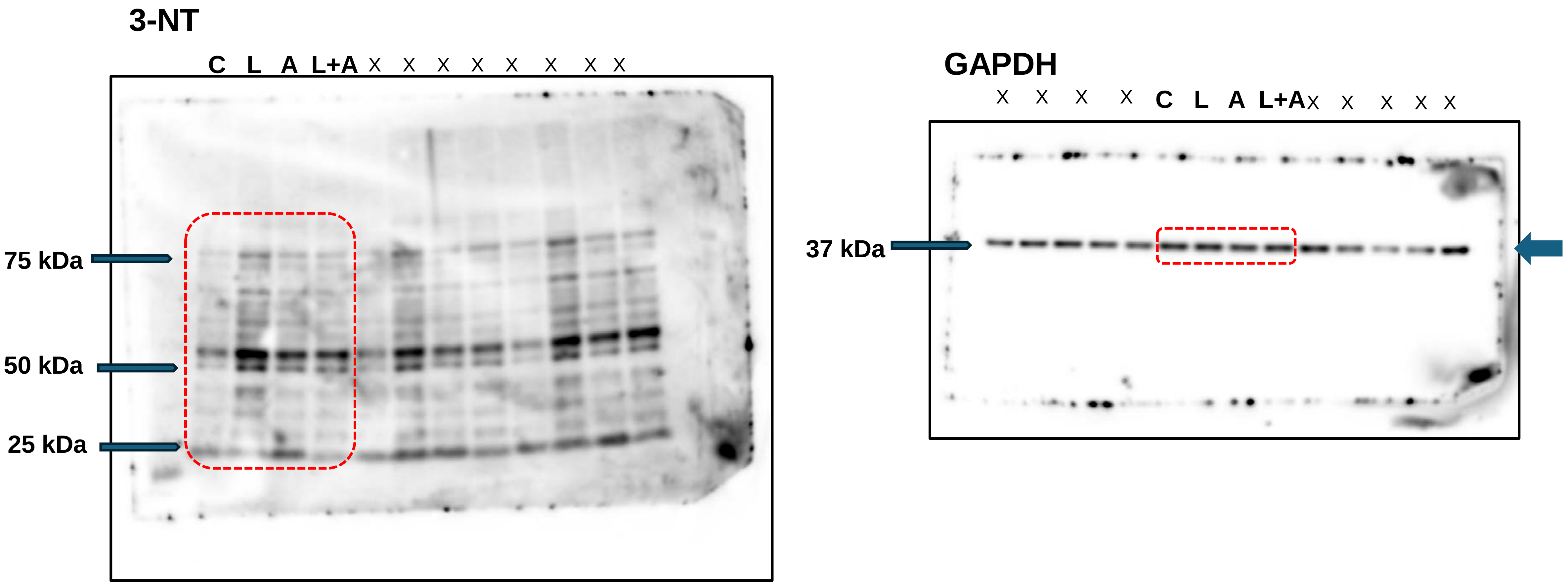

Supplemental Figure 8
Representative full-length immunoblots of **Fig 5D**. The amounts of 3-NT(left panel) and GAPDH (right panel) are shown. The box outlined in red in each panel, indicated by the arrow, corresponds to the cropped part of the blot. C: control, L: PG-LPS, A: allopurinol, L+A: PG-LPS + allopurinol

Supplemental Figure 9

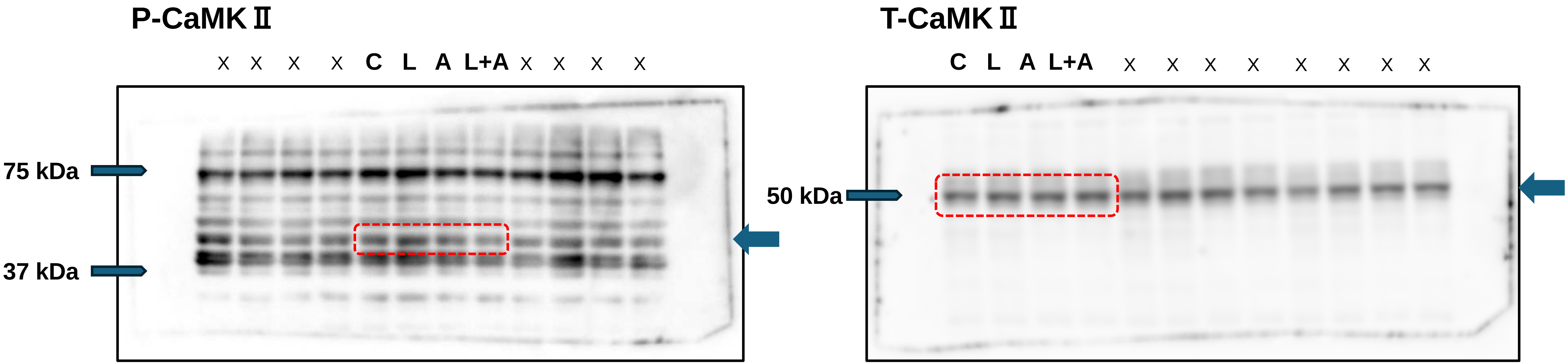

Supplemental Figure 9

Representative full-length immunoblots of **Fig 6A**. The amounts of phospho-CaMKII (Thr-286) (left panel) and total CaMKII (right panel) are shown. The box outlined in red in each panel, indicated by the arrow, corresponds to the cropped part of the blot. C: control, L: PG-LPS, A: allopurinol, L+A: PG-LPS + allopurinol

Supplemental Figure 10

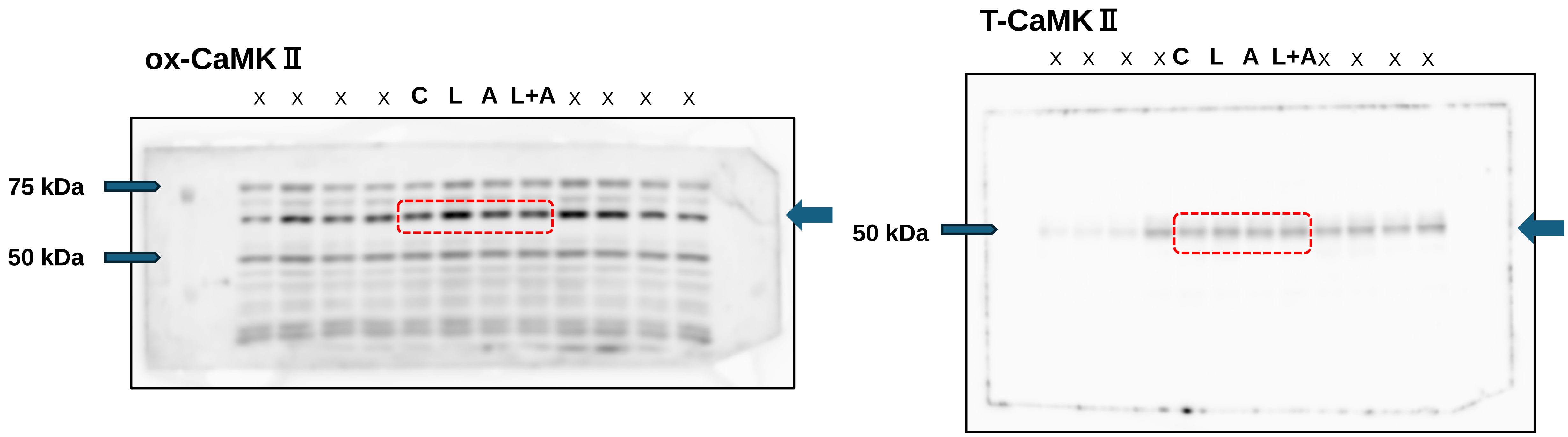

Supplemental Figure 10
Representative full-length immunoblots of **Fig 6B**. The amounts of activated oxidized CaMKII (ox-CaMKII) (left panel) and total CaMKII (right panel) are shown. The box outlined in red in each panel, indicated by the arrow, corresponds to the cropped part of the blot. C: control, L: PG-LPS, A: allopurinol, L+A: PG-LPS + allopurinol

Supplemental Figure 11

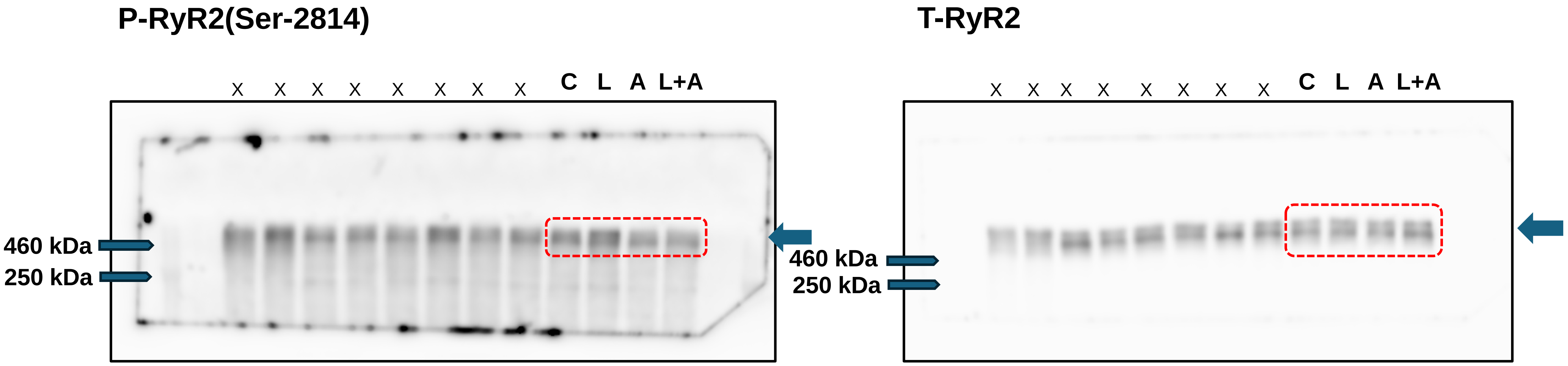

Supplemental Figure 11
Representative full-length immunoblots of **Fig 6C**. The amounts of phospho-RyR2 (Ser-2814) (left panel) and total RyR2 (right panel) are shown. The box outlined in red in each panel, indicated by the arrow, corresponds to the cropped part of the blot. C: control, L: PG-LPS, A: allopurinol, L+A: PG-LPS + allopurinol

Supplemental Figure 12

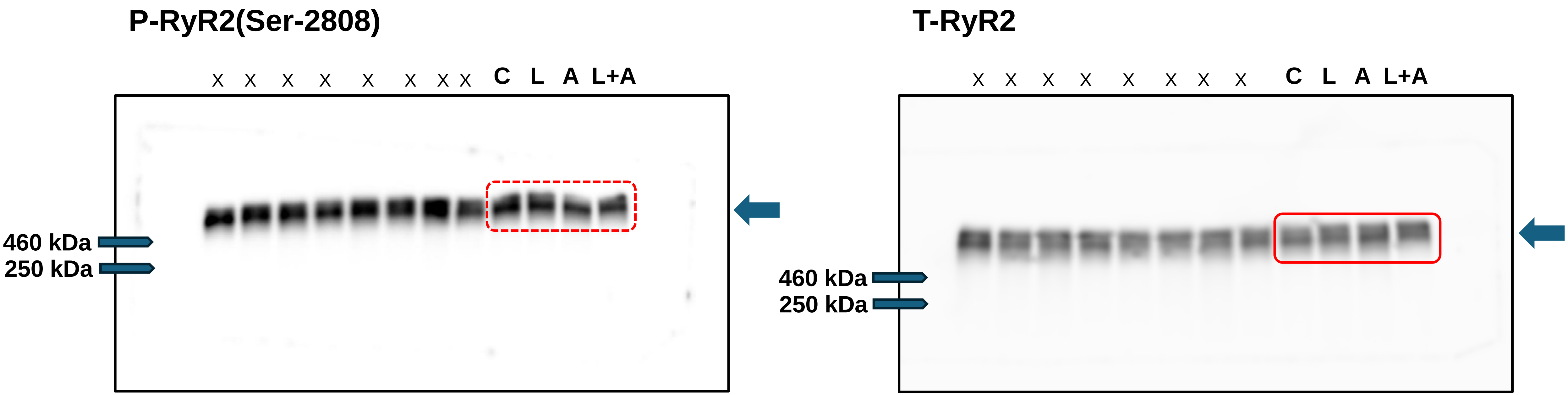

Supplemental Figure 12

Representative full-length immunoblots of **Fig 6D**. The amounts of phospho-RyR2 (Ser-2808) (left panel) and total RyR2 (right panel) are shown. The box outlined in red in each panel, indicated by the arrow, corresponds to the cropped part of the blot. C: control, L: PG-LPS, A: allopurinol, L+A: PG-LPS + allopurinol

Supplemental Figure 13

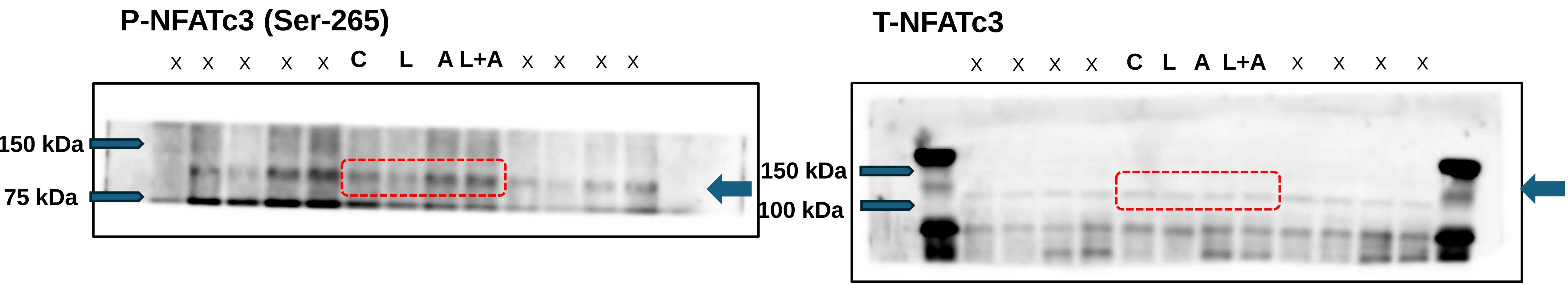

Supplemental Figure 16

Representative full-length immunoblots of **Fig 6E**. The amounts of phospho-NFATc3 (Ser-265) (left panel) and total NFATc3 (right panel) are shown. The box outlined in red in each panel, indicated by the arrow, corresponds to the cropped part of the blot. C: control, L: PG-LPS, A: allopurinol, L+A: PG-LPS + allopurinol
