# Supplementary material for: Allopurinol attenuates development of Porphyromonas gingivalis LPS-induced cardiomyopathy in mice
Source: PLoS One. 2025 Apr 3;20(4):e0318008. doi: 10.1371/journal.pone.0318008 (PMC11967946; doi:10.1371/journal.pone.0318008)
Supplement: S2 Data — (PDF) [file pone.0318008.s002.pdf]

S2 Data

Allopurinol attenuates development of *Porphyromonas gingivalis* LPS-induced cardiomyopathy in mice

Running title: Oxidative heart stress in periodontitis

Akinaka Morii [1, 2 ¶], Ichiro Matsuo [2, 3¶], Kenji Suita [1], Yoshiki Ohnuki [1], Misao Ishikawa [4], Aiko Ito [5], Go Miyamoto [1, 5], Mariko Abe [1, 5], Takao Mitsubayashi [1, 5], Yasumasa Mototani [1], Megumi Nariyama [6], Ren Matsubara [1, 6], Yoshio Hayakawa [7], Yasuharu Amitani [8], Kazuhiro Gomi [2], Takatoshi Nagano [2], Satoshi Okumura [1*]

[1] Department of Physiology, Tsurumi University School of Dental Medicine, Yokohama 230-8501, Japan
[2] Department of Periodontology, Tsurumi University School of Dental Medicine, Yokohama 230-8501, Japan
[3] Department of Oral and Maxillofacial Surgery, Ibaraki Medical Center Tokyo Medical University, Ibaraki 300-0395, Japan
[4] Department of Oral Anatomy, Tsurumi University School of Dental Medicine, Yokohama 230-8501, Japan
[5] Department of Orthodontology, Tsurumi University School of Dental Medicine, Yokohama 230-8501, Japan
[6] Department of Pediatric Dentistry, Tsurumi University School of Dental Medicine, Yokohama 236-8501, Japan
[7] Department of Dental Anesthesiology, Tsurumi University School of Dental Medicine, Yokohama 230-8501, Japan
[8] Department of Mathematics, Tsurumi University School of Dental Medicine, Yokohama, Japan

Supplemental Figure 1

	C	L	A	L+A
n	6	7	6	7
Food (g/day)	9.0 ± 0.7	8.9 ± 1.2	8.8 ± 1.0	9.2 ± 1.1
Water (ml/day)	2.4 ± 0.4	2.2 ± 0.6	2.7 ± 1.1	2.8 ± 0.5

Supplemental Figure 1
Consumed amounts of food and water were similar among the Control (n = 6), PG-LPS (n = 7), allopurinol (n = 6) and PG-LPS + allopurinol (n = 7). P = NS vs. Control). Data shows means ± SD, NS, not significantly different from the Control ($P > 0.05$) by one-way ANOVA followed by the Tukey-Kramer *post hoc* test. C: control, L: PG-LPS, A: allopurinol, L+A: PG-LPS + allopurinol

Supplemental Figure 2

TUNEL Negative control Positive control

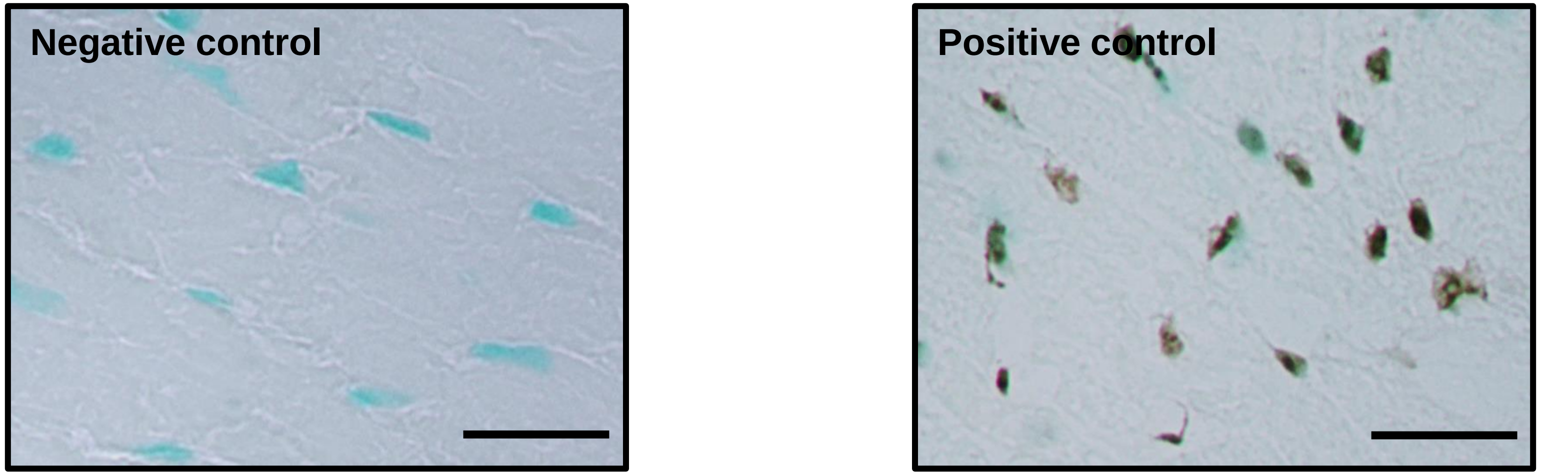

Supplemental Figure 2

Representative images of negative (left) and positive (right) controls of TUNEL staining. Scale bar: 2 μm

Supplemental Figure 3

8-OHdG Negative control Positive control

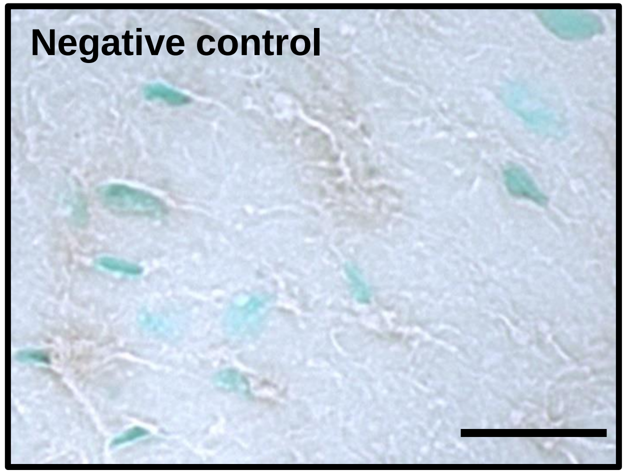

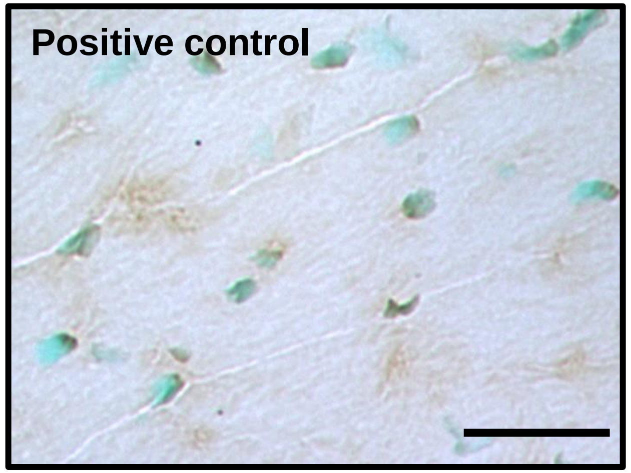

Supplemental Figure 3
Representative negative (left) and positive (right) controls of 8-OHdG staining. Scale bar: 2 μm
