# Supplementary material for: Allopurinol attenuates development of Porphyromonas gingivalis LPS-induced cardiomyopathy in mice
Source: PLoS One. 2025 Apr 3;20(4):e0318008. doi: 10.1371/journal.pone.0318008 (PMC11967946; doi:10.1371/journal.pone.0318008)
Supplement: Attachment — Submitted filename: PLOS ONE Response Revised-01-add RS correction-02.pdf [file pone.0318008.s006.pdf]

<u>Reviewer #1:</u>

The authors have already revised the manuscript which meets my previous requirements. But there are still some minor problems as outlined below.

1. The figure title of Fig5 and Fig6 are the same.

Response-1: Thank you for pointing this out. We modified the title of Fig 6 as shown below.

Effects of allopurinol on PG-LPS-induced phospho-CaMKII, ox-CaMKII, phospho-RyR2 (Ser-2814), phospho-RyR2 (Ser-2808) and phospho-NFATc3 in cardiac muscle.

2. In Fig7, there's an arrow from allopurinol to ROS, and it appears that allopurinol increases ROS production which contradicts the findings. The authors need to modify the schematic diagram to make it more intuitive for the readers.

Response-2: We deleted the line from allopurinol to ROS in Fig 7. Instead, we drew new lines from NOX4 to ROS and XO to ROS, which should clarify the situation for readers.

3. In the legend of Fig7, the authors focused on describing their previous discovery without mentioning their finding that allopurinol can protect heart from PG-LPS induced cardiac dysfunction.

Response-3: We modified the figure legend as shown below to include this information.

Schematic illustration of the proposed role of XO and NOX4 in the heart of PG-LPS-treated mice.

PG-LPS induces expression of XO and NOX4, leading to ROS production, which mediates CaMKII activation and RyR2 phosphorylation (Ser-2814). We previously demonstrated that PG-LPS might induce myocardial ROS production and Ca^{2+}-mishandling via activation of the RAS [1] and cAMP/PKA signaling [2]. Our current study indicates that allopurinol might have a protective effect against PG-LPS-mediated cardiac dysfunction by blocking the increase of ROS generation by XO and NOX4 and Ca^{2+} leakage via altered RyR2 phosphorylation in mice. Solid black

lines represent findings in this study and solid gray lines represent findings reported previously [1, 2]. RAS; renin-angiotensin-aldosterone system; β-AR, β-adrenergic receptor; SR, sarcoplasmic reticulum; RyR2, ryanodine receptor 2; AT1, angiotensin II type 1 receptor; PKA, protein kinase A; ROS, reactive oxygen species; cAMP, cyclic AMP

Reviewer #2:

The authors properly acknowledged the concerns raised in my review. They improved considerably the quality of the text with the figures. I am glad to recommend the acceptance of this manuscript for publication.

Response: Thank you.

References

1. Kiyomoto K, Matsuo I, Suita K, Ohnuki Y, Ishikawa M, Ito A, et al. Oral angiotensin-converting enzyme inhibitor captopril protects the heart from Porphyromonas gingivalis LPS-induced cardiac dysfunction in mice. PloS one. 2023;18(11):e0292624. https://doi: 10.1371/journal.pone.0292624. PMID: 37983238.

2. Matsuo I, Ohnuki Y, Suita K, Ishikawa M, Mototani Y, Ito A, et al. Effects of chronic *Porphylomonas gingivalis* lipopolysaccharide infusion on cardiac dysfunction in mice. J Oral Biosci. 2021;63(4):394-400. https://doi: 10.1016/j.job.2021.10.001. PMID: 34757204.
